# *Opuntia ficus-indica* (L.) Mill. - anticancer properties and phytochemicals: current trends and future perspectives

**DOI:** 10.3389/fpls.2023.1236123

**Published:** 2023-10-04

**Authors:** Jiao Wang, Neeraj Rani, Seema Jakhar, Rakesh Redhu, Sanjiv Kumar, Sachin Kumar, Sanjeev Kumar, Bhagwati Devi, Jesus Simal-Gandara, Bairong Shen, Rajeev K. Singla

**Affiliations:** ^1^ Joint Laboratory of Artificial Intelligence for Critical Care Medicine, Department of Critical Care Medicine and Institutes for Systems Genetics, Frontiers Science Center for Disease-related Molecular Network, West China Hospital, Sichuan University, Chengdu, China; ^2^ Shri Baba Mastnath Institute of Pharmaceutical Science and Research, Baba Mastnath University, Asthal Bohar Rohtak, Haryana, India; ^3^ Department of Pharmaceutical Sciences, Chaudhary Bansi Lal University, Bhiwani, Haryana, India; ^4^ Geeta Institute of Pharmacy, Geeta University, Panipat, Haryana, India; ^5^ Universidade de Vigo, Nutrition and Bromatology Group, Analytical Chemistry and Food Science Department, Faculty of Science, Ourense, Spain; ^6^ School of Pharmaceutical Sciences, Lovely Professional University, Phagwara, Punjab, India

**Keywords:** cancer, *Opuntia ficus-indica*, prickly pear, antioxidant, phytochemicals

## Abstract

Cancer is a leading cause of mortality worldwide, and conventional cancer therapies such as chemotherapy and radiotherapy often result in undesirable and adverse effects. Natural products have emerged as a promising alternative for cancer treatment, with comparatively fewer side effects reported. *Opuntia ficus-indica* (L.) Mill., a member of the *Cactaceae* family, contains a diverse array of phytochemicals, including flavonoids, polyphenols, betalains, and tannins, which have been shown to exhibit potent anticancer properties. Various parts of the Opuntia plant, including the fruits, stems/cladodes, and roots, have demonstrated cytotoxic effects against malignant cell lines in numerous studies. This review comprehensively summarizes the anticancer attributes of the phytochemicals found in *Opuntia ficus-indica* (L.) Mill., highlighting their potential as natural cancer prevention and treatment agents. Bibliometric metric analysis of PubMed and Scopus-retrieved data using VOSviewer as well as QDA analysis provide further insights and niche to be explored. Most anticancer studies on *Opuntia ficus-indica* and its purified metabolites are related to colorectal/colon cancer, followed by melanoma and breast cancer. Very little attention has been paid to leukemia, thyroid, endometrial, liver, and prostate cancer, and it could be considered an opportunity for researchers to explore *O. ficus-indica* and its metabolites against these cancers. The most notable mechanisms expressed and validated in those studies are apoptosis, cell cycle arrest (G0/G1 and G2/M), Bcl-2 modulation, antiproliferative, oxidative stress-mediated mechanisms, and cytochrome c. We have also observed that cladodes and fruits of *O. ficus-indica* have been more studied than other plant parts, which again opens the opportunity for the researchers to explore. Further, cell line-based studies dominated, and very few studies were related to animal-based experiments. The Zebrafish model is another platform to explore. However, it seems like more in-depth studies are required to ascertain clinical utility of this biosustainable resource *O. ficus-indica*.

## 1 Introduction

Cancer is an uncontrolled division of abnormal cells that can potentially intrude or spread (metastasize) to other body regions (Singla et al., 2022c; Chavda et al., 2023). Cancer is a collection of more than 100 different disorders rather than a single disease. It is an uncontrolled cell division that causes abnormal cell development and spread. These malignant cells may infiltrate other tissues and spread to other body parts (metastasize). Cancer is a collection of over 100 different ailments instead of a single disease. Cancer is the world’s second leading cause of death, accounting for 7.6 million deaths in 2005 (Abbas and Rehman, 2018). Globally, an estimated 11 million people have been diagnosed with cancer, which was expected to climb to 16 million by 2020 (Sarfati et al., 2016). According to estimates, one-third of all new cancer cases may be cured if properly diagnosed and treated (Wang et al., 2013; Ricks, 2015). Chemotherapy is a cancer treatment that is widely used. Because cancer cells lack many of the regulatory processes normal cells have, they continue to divide even when normal cells do not. Chemotherapeutic medicines are more sensitive to cancer cells with this characteristic (Zugazagoitia et al., 2016; Prager et al., 2018). A substantial collection of useful chemotherapeutic drugs has been established after almost five decades of systemic medication research and development. Chemotherapeutics, on the other hand, are not without their own set of issues. Chemotherapeutic treatments can result in a wide range of side effects. 5-fluorouracil, for example, is known to produce myelotoxicity (Klein et al., 2022) and cardiotoxicity (Labianca et al., 1982), and has even been demonstrated to serve as a vasospastic agent, in rare but recorded occurrences (Rastogi et al., 1993). Doxorubicin, another commonly used chemotherapeutic, has been linked to cardiac toxicity (Avilés et al., 1993; Desai et al., 2008; Damiani et al., 2016), renal toxicity (Varela-López et al., 2019), and myelotoxicity (Pourtier-Manzanedo et al., 1995). The toxicity of chemotherapeutic drugs can be a severe problem when treating cancer using allopathy or traditional medicine (Zugazagoitia et al., 2016; Gezici and Şekeroğlu, 2019). Like chemotherapy, natural products also play an essential role in preventing cancer (Rani et al., 2022). Plants consist of various phytochemicals which are responsible for anticancer activity. The structure of natural plant chemicals varies greatly; many are aromatic compounds, most of which are phenols or their oxygen-substituted counterparts. It is beneficial to focus on active phytochemicals for herbal therapy to minimize the adverse effects, as well as pathogenic resistance against antibiotics (Kooti et al., 2017). Plants produce a large number of secondary metabolites that are biosynthetically derived from primary metabolites and are a major source of microbicides, insecticides, and a variety of pharmaceutical medications (Garg et al., 2022; Babbar et al., 2023; Kumar et al., 2023). Medicinal plants or their secondary metabolites have played an essential role in human society for a long time in combating diseases, either directly or indirectly (Greenwell and Rahman, 2015; Sharma et al., 2022). Despite the availability of various synthetic antitumor drugs, researchers are still looking for potent naturally occurring anticarcinogens that can prevent, delay, or reverse cancer progression (Singla et al., 2022b). Plants are essential in cancer treatment (Singla et al., 2021a; Singla et al., 2022a). Plant-derived chemicals are thought to account for more than half of all anticancer drugs (Omara et al., 2020). Plant extracts were employed to cure a variety of ailments, and this is the foundation of all Indian medical systems (Singla et al., 2021b). However, compared to modern medicine, this subject is underdeveloped owing to a lack of scientific documentation in this field (Chanchal et al., 2017).

Natural foods and food-derived antioxidants, such as vitamins and phenolic phytochemicals, have recently gotten much attention because they protect against oxidative damage and genotoxicity. During the last 20 years, demand for fresh and ready-to-eat items has increased interest in minimally processed fruits and vegetables, which combine freshness and convenience (Kim et al., 1993). Plant forms have long been known to provide medical benefits (Pandey et al., 2023). This review comprehensively summarizes the anticancer attributes of the phytochemicals found in *Opuntia ficus-indica* (L.) Mill. Several species of cactus pear plants (*Cactaceae* family) evolved in Central America (Mexico). In folk medicine, Opuntia fruits and young stems have long been used to treat diseases such as hypertension, diabetes, allergies and asthma, burns, swelling, and nausea. (Abou-Elella and Ali, 2014). The most significant bioactive substances found in cactus fruit are betaxanthin, betacyanin, and phenolic compounds, which all have potent antioxidant capabilities (Gentile, 2004; Kuti, 2004). Phenolic compounds, especially their effective variants, have an aromatic ring bearing one or more hydroxyl groups. Both chemical compositions and concentrations vary considerably depending on the kind of plant tissue, variety, and ripening phases. *Cactaceae* plants and fruits have been shown to contain glycosylated flavonols, dihydroflavonols, flavonones, and flavonols (Kuti, 2000). Cactus pear fruit is a crucial product for protecting human health against degenerative diseases like cancer, diabetes, hyperglycemia, hypercholesterolemia, arteriosclerosis, and gastric ailments because of the phenolic compounds’ antioxidant qualities (Galati et al., 2003). *O. ficus-indica*’s polyphenolic chemicals have been demonstrated to cause the plasma membrane to become hyperpolarized and to increase the intracellular calcium pool in human Jurkat T-cell strains (Aires et al., 2004).

## 2 Methodology

An extensive survey of the chemical composition and pharmacological activities of *Opuntia ficus-indica* (L.) Mill. was conducted in scientific databases, including Research Gate, Pubmed, Scopus, Science Direct, Web of Science, and Google Scholar. The search terms “*Opuntia ficus-indica*,” “*Opuntia ficus-indica* and biological activity,” “*Opuntia ficus-indica* and pharmacological activity,” “*Opuntia ficus-indica* and chemical compounds,” and “*Opuntia ficus-indica* and chemical profile,” were used for data collection. One hundred and ten publications were included, from 1992 to 2022, from the articles focused on phytochemicals obtained from *O. ficus-indica*.

## 3 *Opuntia ficus-indica* (*O. ficus-indica*)- ethnopharmacological perspectives

**Figure 1:**
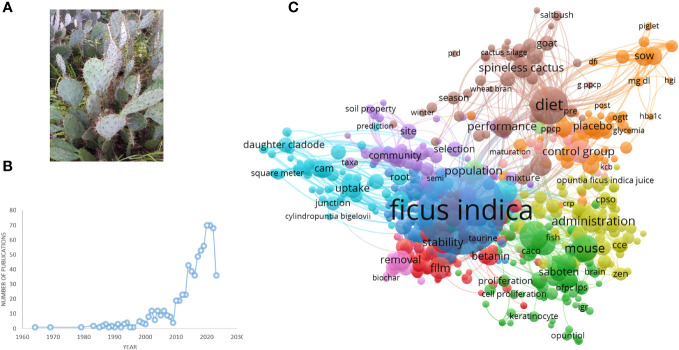
**(A)** *Opuntia ficus-indica*. **(B)** Number of publications related to *Opuntia ficus-indica* over the period until 02.06.2023, as indicated in PubMed. **(C)** Term interaction map based on the literature related to *Opuntia ficus-indica* (the data obtained from PubMed dated 02.06.2023 and processed in VOSviewer).

*O. ficus-indica* (L.) Mill., sometimes known as the prickly pear or nopal cactus, pertains to the *Cactaceae* family of dicotyledonous angiosperms, which comprises around 1500 species of cactus (**Figure 1A**). The plant *O. ficus-indica* is native to the tropics and subtropics (Abou-Elella and Ali, 2014). It can grow in arid and semi-arid environments and can be found in South Africa, Mexico, Latin America, and the Mediterranean region (El-Mostafa et al., 2014). Moroccan inhabitants now recognize three kinds of *O. ficus-indica*. The first is known as “Christians’ nopal” and is widely employed as a field barrier. It is made up of thorny cladodes. The second, cladodes of inermis, is known as “Muslims’ nopal” and is used as green feed for cattle. The final variation, “Moses’ nopal,” has huge inermis cladodes and produces a gigantic pear. It grows primarily in the south of Morocco (Madrigal-Santillán et al., 2022). The juice, jam, oil, and tea derived from the prickly pear of the nopal cactus are used in health, nutrition, and cosmetics (El-Mostafa et al., 2014). Indigenous peoples eat large amounts of fresh or dried fruits. Cactus cladodes, fruits, and flowers are highlighted in these populations due to their high antioxidant, pectin polysaccharide, and fiber content (Farag et al., 2020).

In several nations, it is utilized as an natural treatment for various health conditions (Slimen et al., 2017). Fresh or dried fruits are consumed in large quantities by indigenous people. Cactus cladodes, fruits, and flowers are promoted in these populations due to their pectin polysaccharide, high antioxidant, and fiber content (Lu et al., 2019). Recent scientific studies have emphasized the synthesis of many bioactive molecules that promote *O. ficus-indica* (L.) Mill medicinal uses and pharmacological characteristics. These molecules include carbohydrates, minerals, amino acids, fatty acids, vitamins, fibers, and secondary metabolites recognized for their antioxidant and anticancer properties (Trachtenberg and Mayer, 1981). The primary aim of this article is to summarize and emphasize the benefits of *O. ficus-indica* in terms of cancer prevention and treatment (Park and Chun, 2001).

## 4 *Opuntia ficus-indica* as medicinal and nutritional plant: a bibliometric analysis

We have searched PubMed using the search term “*Opuntia ficus-indica*,” and 628 articles were retrieved as of 02.06.2023 (**Figure 1B**), which covered 19 clinical trials articles, three metanalysis articles, 15 randomized controlled trial-based articles, 31 review articles, and four systematic reviews. Some of the countries that researched more on the topics oriented towards *Opuntia ficus-indica* are Italy (98), Brazil (38), Korea (37), Spain (37), France (35), Germany (26), South Africa (17), India (13), China (7), United States (5), Australia (5), and United Kingdom (5). When we processed the title and abstracts of those 628 articles in the VOSviewer, it yielded 16411 keywords. Once we set criteria for a minimum occurrence of a term five times, it resulted in 1229 terms. For each of the 1229 terms, a relevance score will be calculated and based on the score, and with a cutoff of 60% most relevant terms, it finally yielded 737 terms out of 628 articles. The top 10 keywords are given in **Table 1**, and the overall interactions of all the keywords are illustrated in **Figure 1C**. The bigger the bubble is, the more it is dominant as keywords in the searched literature.

**Table 1:** Top 10 keywords as retrieved by VOSviewer from the publications data related to *Opuntia ficus-indica* obtained from PubMed.

Keywords	Occurrences	Relevance
Gardenia seed	5	3.09
Green tea	6	2.93
Sc co	7	2.72
Cactus pear polysaccharide	5	2.71
Ice plant	6	2.68
Nhdf	5	2.57
Metabolic activity	6	2.48
Fibroblast	9	2.16
Susceptibility	5	2.14
M pulegium	5	2.11

**Figure 2:**
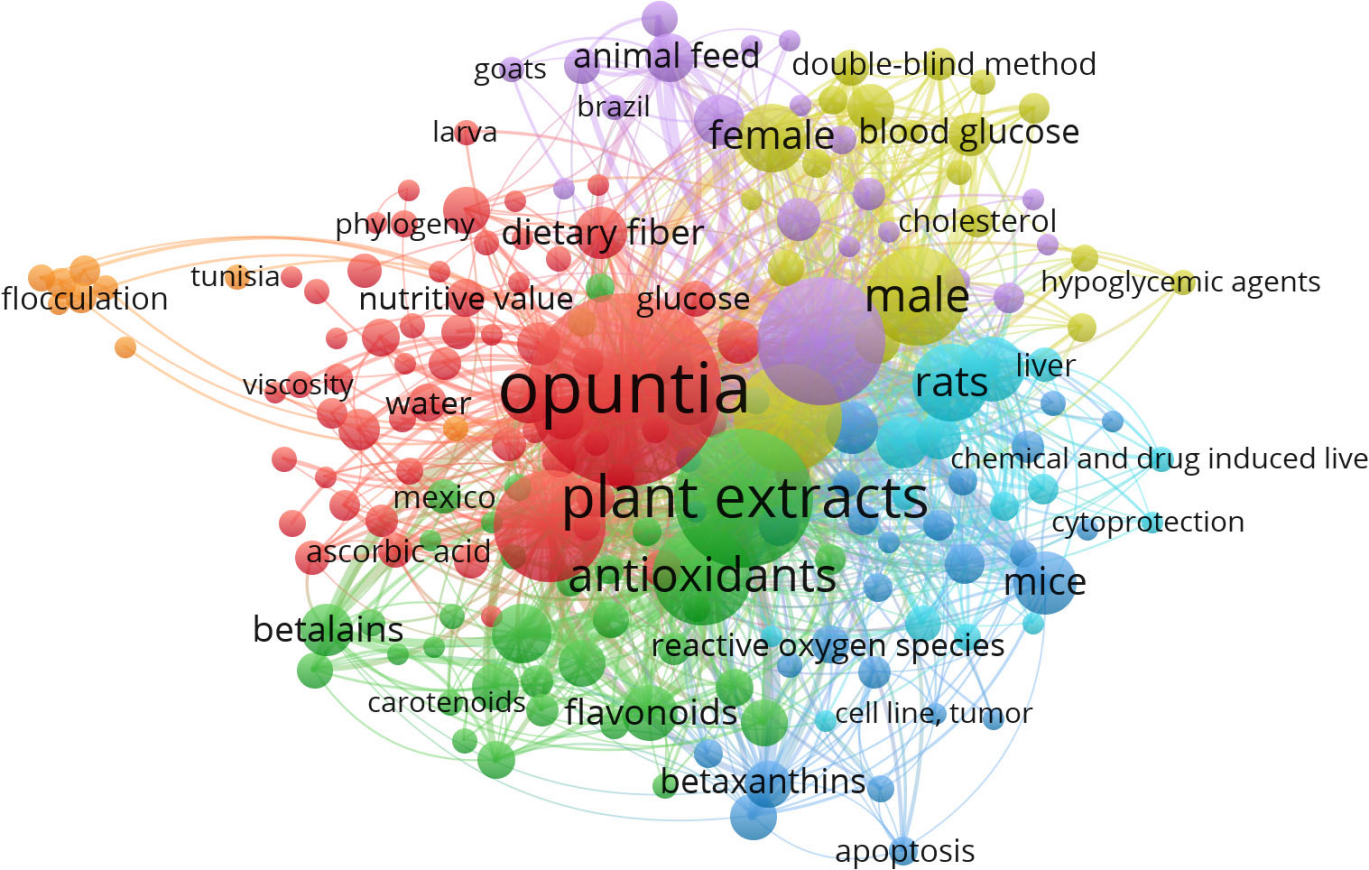
MeSH Term interaction map based on the literature related to *Opuntia ficus-indica* (the data obtained from PubMed dated 02.06.2023 and processed in VOSviewer).

Nevertheless, we have observed only 1375 MeSH terms and 189 terms that have minimally appeared five times in the selected literature. The top 10 MeSH terms are given in **Table 2**, and the overall MeSH term map is illustrated in **Figure 2**. We could see the prevalence of work on *Opuntia ficus-indica* related to apoptosis, cell line tumor, antioxidants, cytoprotection, and reactive oxygen species. It reveals the strong potential of *Opuntia ficus-indica* as a therapeutic reservoir and further encourages us to explore the anticancer potential of *Opuntia ficus-indica.*

**Table 2:** Top 10 MeSH keywords as retrieved by VOSviewer from the publications data related to *Opuntia ficus-indica* obtained from PubMed.

MeSH Keywords	Occurrences	Total Link Strength
Opuntia	352	2255
Plant extracts	182	1364
Animals	154	1184
Male	94	850
Fruit	118	818
Humans	112	817
Antioxidants	94	800
Rats	57	549
Rats, Wistar	41	417
Female	45	360

## 5 *Opuntia ficus-indica* with anticancer potential: a bibliometric analysis

**Figure 3:**
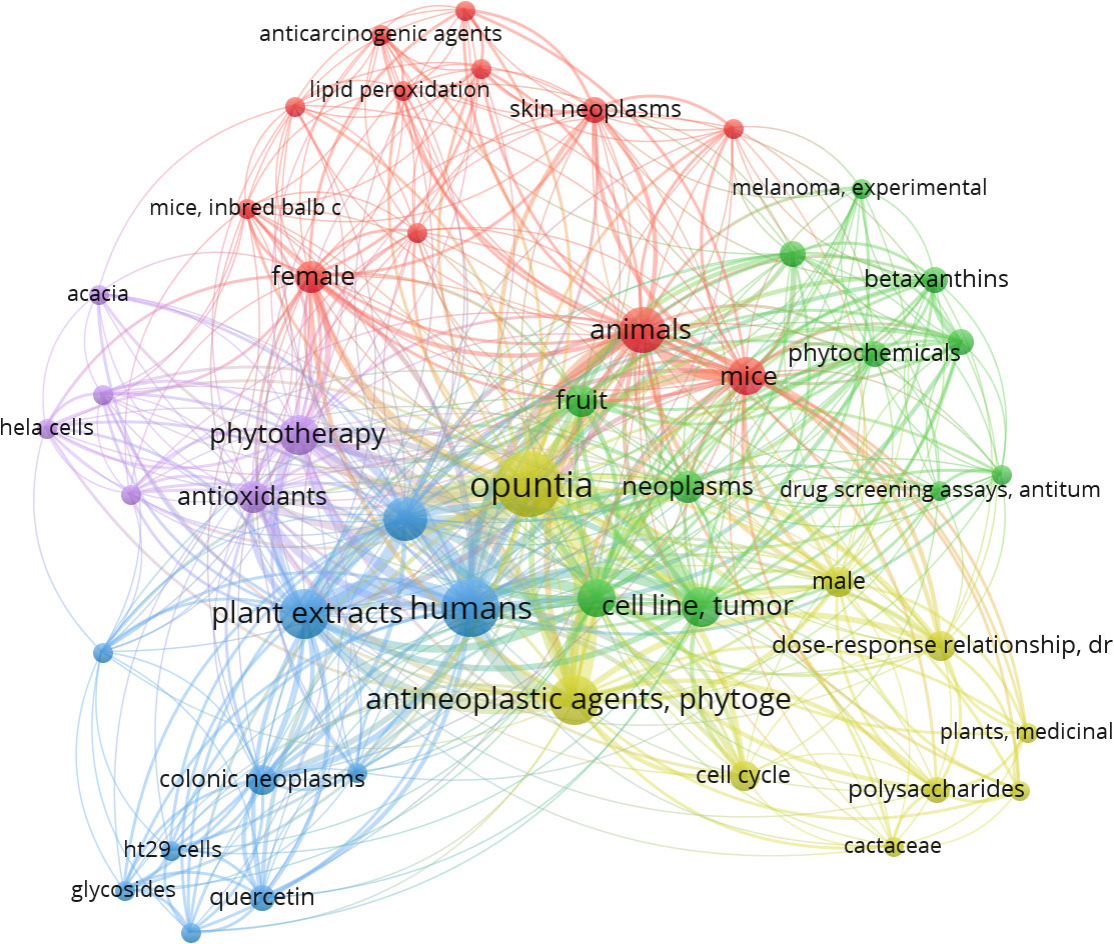
MeSH terms interaction map based on the literature on *Opuntia ficus-indica* and cancer (the data obtained from PubMed dated 01.08.2023 and processed in VOSviewer).

When the MeSH terms “*Opuntia ficus-indica*” and “Cancer” were searched on PubMed (dated 01.08.2023), it resulted in 23 articles, including one review article. When the publication data was processed in VOSviewer, it yielded 157 MeSH terms in the title and abstract of these articles. 48 MeSH terms have been repeated in a minimum of 2 publications. **Figure 3** is the interactive mapping between these 48 MeSH terms. The most co-occurred MeSH terms were “opuntia,” “humans,” “antineoplastic agents, phytogenic,” “plant extracts,” “animals,” “apoptosis,” “phytotherapy,” “cell line, tumor,” “cell proliferation,” and “mice.” We could also observe other vital terms like skin neoplasms, melanoma, colonic neoplasms, HeLa cells, and many others.

**Figure 4:**
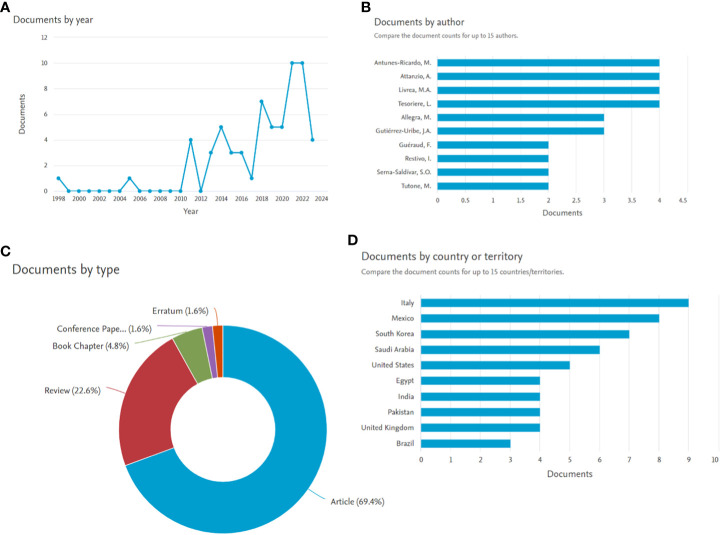
Bibliometric analysis of manuscript published with search terms *Opuntia ficus-indica* and cancer (the data obtained from Scopus dated 01.08.2023). **(A)**: Documents by year; **(B)**: Documents by author; **(C)**: Documents by type; **(D)**: Documents by country or territory.

While exploring Scopus for the articles published with the terms “*Opuntia ficus-indica*” and “cancer,” present in Title/Abstract/Keywords, yielded 62 documents. The analyze tool embedded within Scopus was further utilized to do the bibliometric analysis (**Figure 4**). It has been observed that in the past five years, publications related to “*Opuntia ficus-indica*” and “cancer” have increased significantly (**Figure 4A**). Four research groups “Antunes-Ricardo, M. et al.,” “Attanzio, A. et al.,” “Livrea, M.A. et al.,” and “Tesoriere, L. et al.,” have published four papers each (**Figure 4B**). Top-most cited article from Antunes-Ricardo, M. et al. was “Induction of Apoptosis in Colon Cancer Cells Treated with Isorhamnetin Glycosides from *Opuntia ficus-indica* Pads” with 71 citations as of date (Antunes-Ricardo et al., 2014). The top-most cited article from Attanzio, A. et al., Livrea, M.A. et al., and Tesoriere, L. et al. was the same. It was entitled “Antiproliferative and pro-apoptotic activity of whole extract and isolated indicaxanthin from *Opuntia ficus-indica* associated with re-activation of the onco-suppressor p16INK4a gene in human colorectal carcinoma (Caco-2) cells” with 50 citations as on date (Naselli et al., 2014). Of 62 documents, 69.4% were articles, followed by 22.6% review articles (**Figure 4C**), and Italy was recorded as the country with the highest number of documents, followed by Mexico and South Korea (**Figure 4D**).

**Figure 5:**
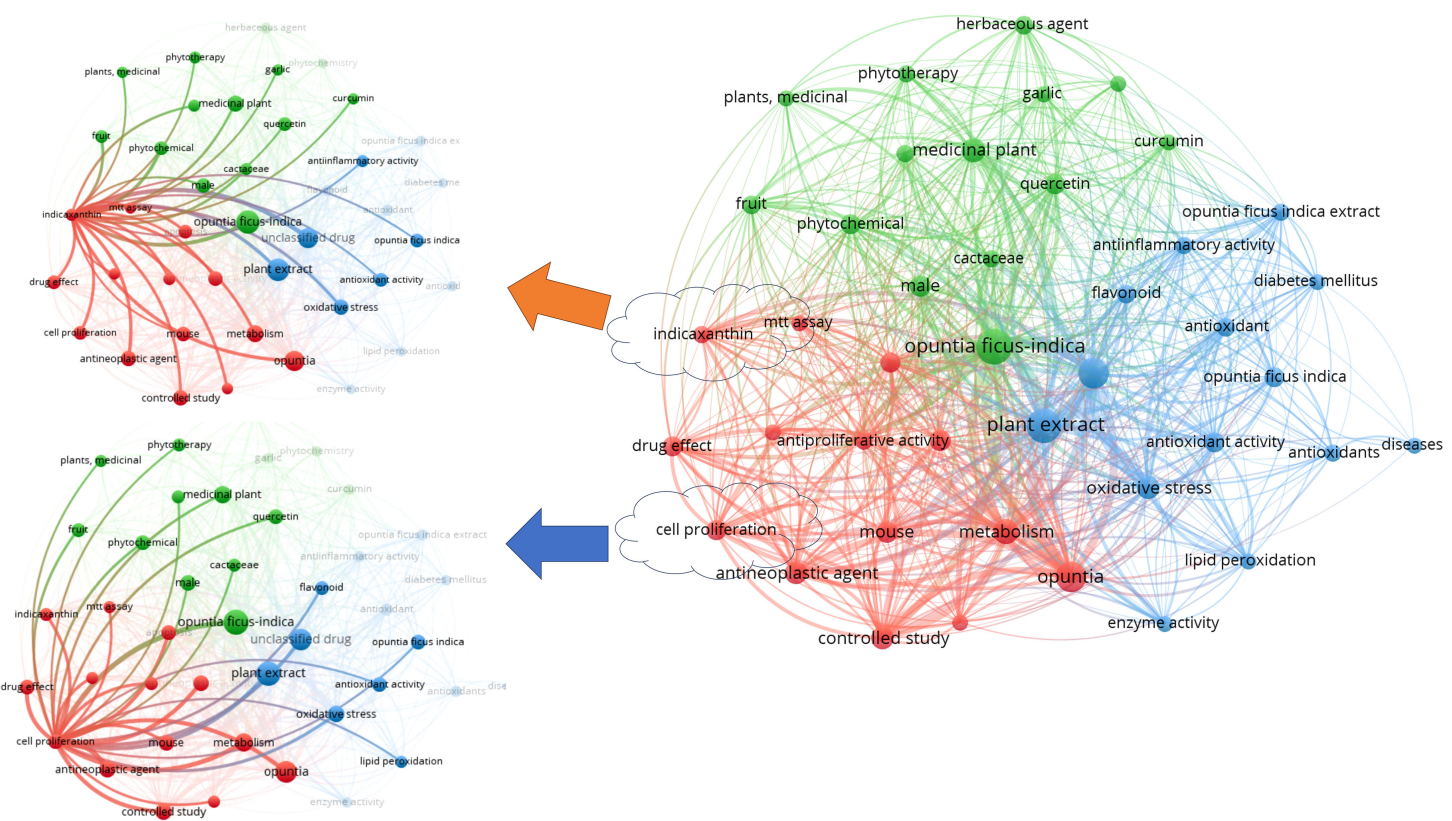
Indexed keywords interaction map based on the literature related to *Opuntia ficus-indica* and cancer (the data obtained from Scopus dated 01.08.2023 and processed in VOSviewer).

When the bibliometric data from these 62 Scopus-retrieved documents were extracted and imported in VOSviewer, there were 1891 indexed keywords. Of these 1891 indexed terms, 59 are visible in at least five publications. Before analysis, some indexed keywords were manually removed like “human,” “humans,” “nonhuman,” “chemistry,” “article,” “plant extracts,” “animal,” “animals,” “priority journal,” “review,” “isolation and purification,” “human cell,” “animal experiment,” “animal tissue,” “mice,” “drug mechanism,” and “procedures.” **Figure 5** illustrates the interactive mapping between the selected indexed keywords retrieved from these 62 documents. Along with the *Opuntia ficus-indica* and cancer-specific terms, important co-occurring terms were oxidative stress, antioxidants, anti-inflammatory activity, and diabetes mellitus. Indicaxanthin was strongly connected with cell proliferation and antineoplastic agents. Cell proliferation seems to strongly associate with oxidative stress and lipid peroxidation as a co-occurred term.

## 6 Role of *Opuntia ficus-indica* in cancer treatment and management

In several studies, abundant Opuntia parts, including prickly pear fruits, seeds, peels, stems, cladodes, and roots, have been shown to have cytotoxic effects on malignant cell lines (**Table 3**). Antunes-Ricardo et al. (Antunes-Ricardo et al., 2014) investigated the cytotoxic activity of purified isorhamnetin glycosides or cladode flour extracts of *O. ficus-indica* (var. Jalpa) on two types of cancer cell lines of the human colon, Caco-2, and HT-29, which represent apoptosis-resistant and apoptosis-susceptible cell lines, successively, with normal fibroblasts (NIH 3T3) were employed as control. According to these authors, the glycosylation pattern of pure isorhamnetin glycosides and extract of cladode flour was more cytotoxic to HT-29 cells than Caco-2 cells. The induction of apoptosis by the caspase cascade, which plays a crucial role in apoptosis pathways, was linked to these effects. Naselli et al. (Naselli et al., 2014) analyzed the results of an aqueous fruit extract of *O. ficus-indica* on the growth of the Caco-2 cancer cell line of the human colon. On proliferating cells, these researchers found no effect on the differentiated cells but a dose-dependent apoptotic effect. This research shows an epigenomic effect on the tumor suppressor gene p16INK4a due to the demethylation of its promoter and stimulation of its expression, indicaxanthin. Betanin, obtained from fruits of *O. ficus-indica*, was found to suppress the development of the human’s chronic myeloid leukemia cell line K562 via the apoptotic intrinsic pathway (Sreekanth et al., 2007).

**Table 3:** Metabolite extracts of *O. ficus-indica* and related species and their anticancer potential.

S. No	The plant part of *O. Ficus-Indica*	Sample Form	Cell lines	Outcome	References
1	Seed	Seed oil	Adeno-carcinoma Cell Lines (Colo-320 and Colo-741)	Reduction in cell viability	(Becer et al., 2018)
2	Stems	Extracts (hexane, ethyl acetate (EtOAc), acetone, methanol (MeOH), and MeOH: water (80:20))	SW480 colon MCF7 breast cancer cells	All except hexane extract exhibited significant cytotoxicity, possibly by COX-2 inhibition and increased Bax/Bcl2 ratio.	(Kim et al., 2015)
3	Fruit	Methanolic extract	U87-MG (glioblastoma multiform; brain cancer) HT-29 (colon cancer) cell lines	Dose-dependent cell death	(Okur et al., 2019)
4	Seed	Seed Oil	Colo-320 Colo-741Colon Carcinoma Cell Lines	Modulates PGE2-mediated and VEGF-dependent angiogenesis	(Becer et al., 2021)
5	Fruit	Aqueous extract	Cancer cell line Caco-2 of the Human colon	Dose-dependent apoptosis	(Naselli et al., 2014)
6	Fruit	Juice	PC3 prostate Caco-2 colon cell lines	The viability of prostate and colon cancer was most affected by juices	(Chavez-Santoscoy et al., 2009)
7	Fruit	Mixture aqueous extract	Ovarian cancer cells (OVCA420, SKOV3)	ROS increase, downregulation of NF-kappaB and p-/SAPK/JNK, and upregulation of p-AKT, the apoptotic effect	(Feugang et al., 2010)
8	Cladode	Powder	Preneoplastic (Apc min/+) immortalized epithelial colon cells	Inhibit LDL oxidation, and increase cytotoxicity	(Keller et al., 2015)
9	Fruit	Alkaline hydrolysis-based extracts	HT-29 and Caco2	More cytotoxic against HT-29 cells than Caco2 cells, increased activity of caspase 3/7	(Antunes-Ricardo et al., 2014)

The toxicity impact from prickly pear fruits-filtered juices of several species of *Opuntia* on various cancer cell lines was investigated by Chavez-Santoscoy et al. (Chavez-Santoscoy et al., 2009). The Caco-2 and PC3 prostate cell lines were most impacted, whereas hepatic HepG2 and mammary gland MCF-7 cell lines grew slower. As a control, normal fibroblasts were used. On cancer cells, *O. rastrera* was the most cytotoxic species, with the highest potential and antioxidant content amongst the distinct species exposed to be tested. Kim et al. demonstrated that cladodes extracts from *O. humifusa* may cause apoptosis in human colon SW-480 and MCF-7 cells (Kim et al., 2015). Water-separated fractions of *O. humifusa* stems and fruits suppressed the development of U87MG glioblastoma cells, which was related to cell proliferation and reactive oxygen species (ROS) production (Hahm et al., 2010). The same researchers reported a similar effect on HeLa cancer cells but not on usual fibroblasts (Hahm et al., 2014). Serra et al. (Serra et al., 2013) reported that juice concentrates rich with polyphenols from several Opuntia were found to be cytotoxic to colorectal cancer cell lines HT- 29 but hardly toxic to Caco-2 and that cell-cycle arrest in the same cells was induced more efficiently by the natural juice remnants extracts (peels and seeds) than juice concentrates. Surprisingly, this effect was associated with a rise in ROS in the cells, suggesting that extracts’ pro-oxidant effects caused cell death induced by ROS. Feugang et al. (Feugang et al., 2010) observed a similar pro-oxidant effect in ovarian cancer cells when compared to immortalized or normal cells. It is essential to use relevant controls, such as cells of the same type with the same genotype, to make conclusions about compounds’ potential benefits. (Phyto)-compounds could be more cytotoxic to cancer cells than their non-cancerous counterparts to be considered as anti-cancer compounds (Keller et al., 2015). Furthermore, various Opuntia cladode flours protect against the cytotoxicity of 4-hydroxynonenal, an oxidation product of dietary lipids that may have a role in promoting red meat for colorectal cancer. Only the usual epithelial cells of the mouse colon showed the protective effect, not on the similar cells that had the Apc type of mutation, whichever is an early phenomenon of colorectal oncogenesis prevalent in humans (Karim and Huso, 2013). Nevertheless, validating the effects seen *in vitro* and *in vivo* studies is essential. Zou et al. observed that aqueous extracts of Opuntia cactus pear inhibited carcinogenesis in nude mice to a similar extent as the synthetic retinamide (4-HPR) retinoid N-(4-hydroxyphenyl) employed as a chemotherapeutic dummy compound (Zou et al., 2005). Hahm et al. demonstrated that *O. humifusa* had a protective effect on HeLa cell xenografts (Hahm et al., 2010). Some researchers noted that cladode extracts of *O. ficus-indica* reduced genotoxicity and oxidative stress caused by the aflatoxin B1 and mycotoxins zearalenone *in vivo* (Zourgui et al., 2008). Extracts of Opuntia were usually administered into the peritoneal cavity. To determine the protective effects of *Opuntia* spp. additional studies are required, including evaluating the compounds by an oral route or in a more physiological condition that takes into consideration the digestion and bioavailability of compounds alike. In such an approach, in two distinct animal models of skin carcinogenesis, lyophilized powder of *O. humifusa* fruit administered through pelletized food was found to be protecting with a decline in inflammation and skin lipid peroxidation (Lee et al., 2012; Lee et al., 2013). All these findings suggest that *Opuntia* spp., whether in the form of fruits, nopal (Opuntia cladodes or stems), or fruit juice, might be an effective anticancer approach.

## 7 Phytochemicals present in *O. ficus-indica* and its anticancer attributes

*Opuntia ficus-indica* is known to have various phytochemicals, which are discussed below.

### 7.1 Polyphenolic compounds

Phenolics are small plant secondary metabolites with at least one hydroxyl group and an aromatic ring (Singla et al., 2019). More than 8000 phenolic chemicals have been found in the vegetable kingdom, with over 4000 flavonoids now known (El Gharras, 2009). Flavonoids are the most frequent phenolic compounds found in plants. They feature a diphenylpropane (C_6_-C_3_-C_6_) core structure with two aromatic rings connected by an oxygenated heterocycle (Harborne and Williams, 2000). The fruits and seeds of *O. ficus-indica* had the lowest concentrations of phenolics and flavonoids, whereas peels, flowers, and cladodes had the highest concentrations. The phenolic profile of the whole Opuntia plant includes 40 phenolic acids, 1 gallotannin, 3 flavanones, 8 flavanols, 18 flavonols, 3 flavononols, and 9 flavones (**Table 4**).

**Table 4:** Phytochemicals and their contents found in different parts of *O. ficus indica*.

**Phytochemicals (unit)**	**Plant Part**	**Content**	**References**
Total phenolics (mg/100gm)	Flower	12022– 27090	(Fernández-López et al., 2010; Jorge et al., 2013; El-Mostafa et al., 2014; Ammar et al., 2015; Ramírez-Ramos et al., 2018)
	Cladodes	390.90	
	Peels	45.700– 425.59	
	Fruits	48.11– 218.8	
	Seeds	48–89	
	Fruit Pulp	5.35 mg GAE/g	(Sigwela et al., 2021)
Total flavonoids (mg/100gm)	Flower	6081– 6267	(Fernández-López et al., 2010; Jorge et al., 2013; El-Mostafa et al., 2014; Ammar et al., 2015; Ramírez-Ramos et al., 2018)
	Cladodes	73	
	Peels	6.95– 23.96	
	Fruits	2.60– 15.560	
	Seeds	1.5– 2.6	
	Fruit pulp	0.63 mg CE/g	(Sigwela et al., 2021)
Total tannins (mg/100gm)	Flower	768.67	(Figueroa-Pérez et al., 2018; Ramírez-Ramos et al., 2018)
	Cladodes	430–620	
	Peels	23– 144	
	Seeds	4.1– 205	
Total anthocyanins (mg/100gm)	Cladodes	0.05– 0.34	(Alves et al., 2017)
Quinic acid (mg/100gm)	Cladodes	42.983– 436.96	(Belhadj Slimen, 2017; Oniszczuk et al., 2020)
	Peels	145.071	
	Fruits	45.471	
Malic acid (mg/100gm)	Cladodes	3124– 4421.7	(Allai et al., 2016)
trans-Aconitic acid (mg/100gm)	Cladodes	77.15– 88.63	(Allai et al., 2016)
Betalains (mg/Kg)	Cladodes	16.17	(De Wit et al., 2020)
	Peels	13.57	
	Fruits	18.52	
β-Sitosterol(mg/Kg)	Cladodes	16.53	(Ramadan and Mörsel, 2003; Allai et al., 2016)
	Peels	21.1	
	Fruits	11.2	
	Seeds	6.75	
	Seed oil	2.80	
Stigmasterol (mg/Kg)	Cladodes	13.4	
	Peels	0.73	
	Fruits	2.12	
	Seeds	0.30	
Δ7-Avenasterol (mg/Kg)	Cladodes	11.6	
	Seeds	0.05	
Campesterol (mg/Kg)	Cladodes	5.7	
	Peels	8.74	
	Fruits	8.76	
	Seeds	1.66	
	Seed oil	0.51	
Campestanol (mg/Kg)	Cladodes	6.4	
Carotenoids (μg/g)	Peels	12.58–16.83	(Fernández-López et al., 2010)
	Fruits	2.58–6.68	
Saponins (g/Kg)	Cladodes	8.72	(Touré et al., 2015)
	Peels	6.36	
	Seeds	20.4	
Ascorbic acid (mg/100 gm)	Fruit pulp	12.35	(Sigwela et al., 2021)

#### 7.1.1 Organic acids

**Figure 6:**
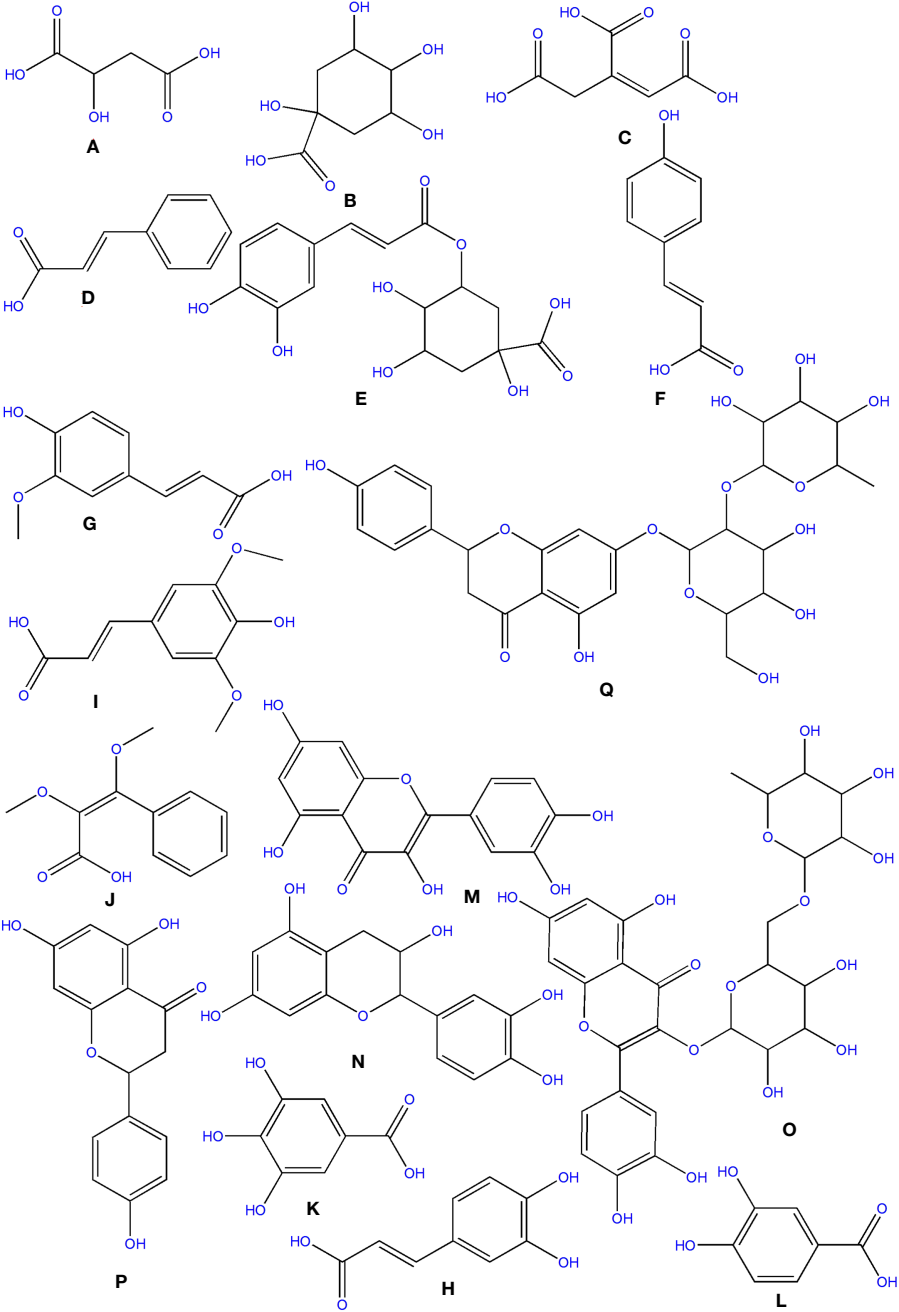
Structure of some of the phytochemicals isolated from *O. ficus-indica*. **(A)** Malic acid; **(B)** Quinic acid; **(C)** Aconitic acid; **(D)** Cinnamic acid; **(E)** Chlorogenic Acid; **(F)** p-Coumaric acid; **(G)** Ferulic acid; **(H)** Caffeic Acid; **(I)** Sinapic acid; **(J)** Dimethoxycinnamic acid; **(K)**: Gallic acid; **(L)** Protocatechuic acid; **(M)** Quercetin; **(N)** Catechin; **(O)** Rutin; **(P)** Naringenin; **(Q)** Naringin.

Three organic acids were discovered in *O. ficus-indica* in addition to the phenolic compounds: malic (**Figure 6A**), quinic (**Figure 6B**), and aconitic (**Figure 6C**) acids. Peels and cladodes had the highest concentrations. More research is required to determine the comparable levels in flowers and seeds. Quinic acid (C_7_H_12_O_6_) is a cyclohexane carboxylic skeleton-containing plant metabolite. Quinic acid is critical in manufacturing aromatic chemicals (flavonoids and phenol carboxylic acids) in higher plants, people, and animals (Bai et al., 2018). D-(-)-Quinic acid has been shown to have antioxidant, anti-inflammatory, and antiproliferative characteristics in addition to its antibacterial activity (Belhadj Slimen et al., 2021). This chemical is also linked to neuro- and radioprotective properties (Liu et al., 2020). Antioxidant, anti-inflammatory, and antiproliferative effects have been described for D-(-)-Quinic acid (Alfieri et al., 2021). In experimental mice, cis-aconitic acid prevented carcinogenesis produced by 3, 4-benzopyrene (Belhadj Slimen et al., 2021).

#### 7.1.2 Phenolic acids

Phenolics are secondary metabolites that have recently attracted attention as anti-cancer agents. Phenolics have much potential as anti-cancer medicines since they promote apoptosis, reduce proliferation, and target several elements of cancer (angiogenesis, growth and differentiation, and metastasis) (Abotaleb et al., 2020). Hydroxybenzoic and hydroxycinnamic acids are different kinds of phenolic acids (Clifford, 1999). Cinnamic acid derivatives include hydroxycinnamic acids. Simple esters containing quinic acid or glucose. Cinnamic (**Figure 6D**), chlorogenic (**Figure 6E**), coumaric (**Figure 6F**), and ferulic (**Figure 6G**) acids are the most prevalent hydroxycinnamic acids in *O. ficus-indica*. Caffeic (**Figure 6H**), sinapic (**Figure 6I**), and dimethoxycinnamic (**Figure 6J**) acids were found in lower quantities (Bai et al., 2018).

##### 7.1.2.1 Gallic acid

Gallic acid (**Figure 6K**), chemically known as 3,4,5-trihydroxybenzoic acid, was discovered in the flowers and cladodes of *O. ficus-indica*. It can be found in plants as an ester, free acid, hydrolyzable tannin, or catechin derivatives. Gallic acid, along with its by-products, has been shown to have a broad range of biological activities like bactericide, antiviral, antifungal, inflammatory harmonizer, antidiabetic activities, and antiproliferative (Kahkeshani et al., 2019). Gallic acid and its derivatives have been shown to have an anti-cancer effect *in vivo* and *in vitro* in several investigations (Sun et al., 2002; Bhattacharya et al., 2016). Gallic acid has been shown to have anti-cancer properties in various cancer cells, including human ovarian cancer cells (Dai and Mumper, 2010; De et al., 2013). Gallic acid’s anti-cancer properties have been proved to be attributable to its capacity to suppress cell proliferation and promote apoptosis (De Mejia et al., 2009).

##### 7.1.2.2 Protocatechuic acid

Protocatechuic acid (PCA) (**Figure 6L**), also known as 3,4-dihydroxybenzoic acid, is a dihydroxybenzoic acid in which the hydroxy groups are attached at positions 3 and 4. Recent research suggests that PCA could be a cancer-fighting agent against neoplasms. Its mode of action is related mainly to its radical scavenging activity, which allows it to limit the formation of free radicals while also up-regulating enzymes involved in the neutralization of radicals (Kakkar and Bais, 2014). PCA has also been described as an anticancer agent and found to be a powerful antioxidant (Belhadj Slimen et al., 2021).

##### 7.1.2.3 Cinnamic acid and derivatives

Cinnamic acid (C_9_H_8_O_2_) is a plant-derived organic acid. It is less toxic and possesses many biological and antioxidant properties (Sova, 2012). Cinnamic acid derivatives have better antioxidant activity than benzoic acid analogs (Natella et al., 1999). Cinnamic acid and its derivatives (natural and non-natural molecules) have been shown to have anticancer potential in recent decades. Various cinnamoyl compounds and their anticancer effectiveness have gotten much interest in recent decades (De et al., 2011) and promote the proliferation of neural progenitor cells (Otero et al., 2014).

##### 7.1.2.4 Chlorogenic acid

Chlorogenic acid (C_16_H_18_O_9_) (CGA), also termed 3-O-caffeoylquinic acid, is a cinnamate ester produced by conventional condensation of the carboxy group of trans-caffeic acid with the 3-hydroxy group of quinic acid. Chlorogenic acid has long been known to be an antioxidant (Meng et al., 2013; Su et al., 2015; Tajik et al., 2017). Its anticarcinogenic, anti-inflammatory, and antioxidant properties might make it a non-invasive cure or preventative approach for some chronic diseases (Tajik et al., 2017). CGA also works as a potent chemo-sensitizing agent, inhibiting cancer growth by activating and inhibiting critical pathways in cancer metabolism (Slimen et al., 2016). CGA’s anticancer molecular processes, on the other hand, are unknown (Abd Elrazik et al., 2019).

##### 7.1.2.5 Coumaric acid and derivatives

**Figure 7:**
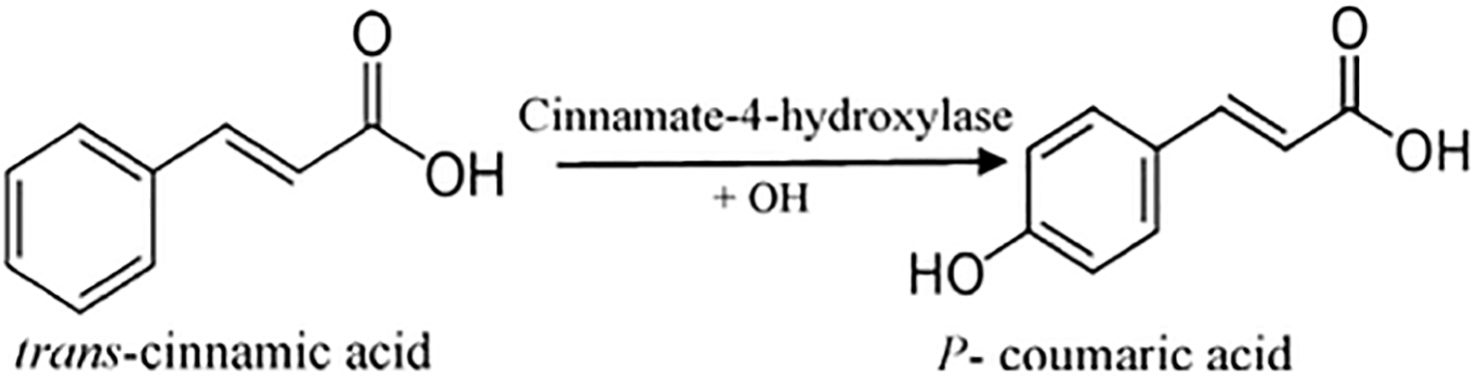
Synthesis of coumaric acid.

P-coumaric acid (C_9_H_8_O_3_), also referred to as 4-hydroxycinnamic acid, is a phenyl ring hydroxylated cinnamic acid analog. It is a conjugate of 4-coumarate. The amino acids tyrosine and phenylalanine are used to synthesize p-coumaric acid. Coumaric acid is crucial in phenolic acid production, including rosmarinic, caffeic, chlorogenic, and ferulic acids. Based on the research by Kiliç and Yeşiloğlu, p-coumaric acid is a reasonable scavenger and potent antioxidant of reactive oxygen species and free radicals (Kiliç and Yeşiloğlu, 2013). It was more efficient than vitamin E in reducing oxidative stress in animal models (Guglielmi et al., 2003). This acid has been drawn to suppress cancer cell growth and migration while promoting apoptotic cancer cell death, indicating that it may have antioxidant effects (Roy et al., 2016). It has also been shown in animal models to have chemopreventive effects against colon cancer. The biosynthesis of coumaric acid is shown in **Figure 7** (Sharma et al., 2018).

##### 7.1.2.6 Ferulic acid

**Figure 8:**
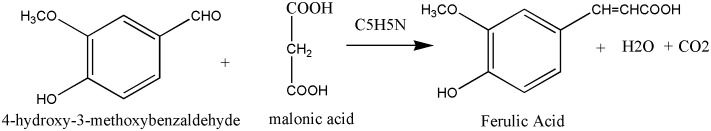
Synthesis of ferulic acid.

Ferulic acid (FA) is a trans-cinnamic acid with hydroxy and methoxy aggregates on the phenyl ring at positions 3 and 4. It is an omnipresent phytochemical occurring in the cell walls of the plant. It may be found in both covalently-coupled and free (rarely) forms, one to lignin and others in biopolymers in leaves and seeds. FA readily generates a resonance-stabilized phenoxyl radical, which gives its high antioxidant activity (Graf, 1992; Mancuso et al., 2007). Ferulic acid has been proven to have anti-cancer properties in a range of cancers, including colon and lung cancers and tumors of the central nervous system. However, its possible effect in limiting breast cancer metastasis is still uncertain (Zhang et al., 2016). Trans-ferulic acid inhibited Hsp60-induced cell growth to a significant extent (Fukuoka et al., 2004). FA has been shown to have anticancer properties. Gao et al. (2018) demonstrated that in Hela and Caski cells, FA can substantially reduce cell invasion and metastasis. Inhibiting autophagy and triggering cell cycle arrest in human cervical carcinoma cells could make it an anti-cancer therapeutic. The synthesis of Ferulic Acid is shown in **Figure 8** (Mancuso and Santangelo, 2014; Gao et al., 2018).

##### 7.1.2.7 Caffeic acid

Caffeic acid (3, 4-dihydroxycinnamic acid) structurally has acrylic and phenolic functional groups (Rocha et al., 2012). It was found in high concentrations in the fruits and flowers of *O. ficus-indica* and was found to have a wide range of biological characteristics. Caffeic acid and its derivatives are effective against oral, colon, and liver cancers and are likely to be cyclooxygenase II inhibitors in malignancies (Slimen et al., 2016). Even though much research has shown caffeic acid’s anticancer activity, some researchers have demonstrated carcinogenic effects, even at modest doses (Hirose et al., 1998). The International Agency for Research on Cancerous disease has categorized this acid as a chemical “possibly carcinogenic to humans” in group 2B. As a result, further research is needed before concluding that caffeic acid has a preventive or carcinogenic potential (Slimen et al., 2016).

#### 7.1.3 Flavonoids

Flavonoids are secondary metabolites derived from plants. They are divided into four primary categories: anthocyanidins, flavanols, flavones, and isoflavonoids. They are also classified into subcategories (Duraipandiyan et al., 2017).

##### 7.1.3.1 Flavanols

Flavanols are a structurally complex subclass of phenolic compounds that range from monomers (such as catechin) to oligomers (from dimers to decamers), polymers, and more complex derivatives (such as theaflavins and thearubigins). Quercetin (**Figure 6M**) and its derivatives, catechin (**Figure 6N**), and rutin (**Figure 6O**), a flavan-3-ol, are the vital flavanols found in *O. ficus-indica*. Although flavanols were not found in Opuntia cladodes and peels, they were in high concentrations in flowers and minimal levels in fruits. Flowers have excessive concentrations of flavanols, followed by cladodes, peels, and fruits. More research is required to determine the flavanol concentration in seeds. Because of its high antioxidant action, rutin is a very potent molecule. It was found to have antifungal, anti-allergic, and antibacterial properties, and it is now used to treat many chronic diseases like diabetes, hypertension, hypercholesterolemia, and cancer. Rutin is a non-oxidizable and non-toxic molecule when compared to other flavonoids (Sharma et al., 2013). Flavonoids have been demonstrated to have a wide range of anti-cancer properties, including the ability to modulate ROS, induce apoptosis, autophagy, invasiveness, and reduce cancer cell growth. Flavonoids have a dual role in ROS homeostasis: they are significant pro-oxidants in cancer cells, initiating apoptosis pathways, and they also act as antioxidants in healthy cells, inhibiting pro-inflammatory signaling pathways (Kopustinskiene et al., 2020).

##### 7.1.3.2 Flavanones

**Figure 9:**
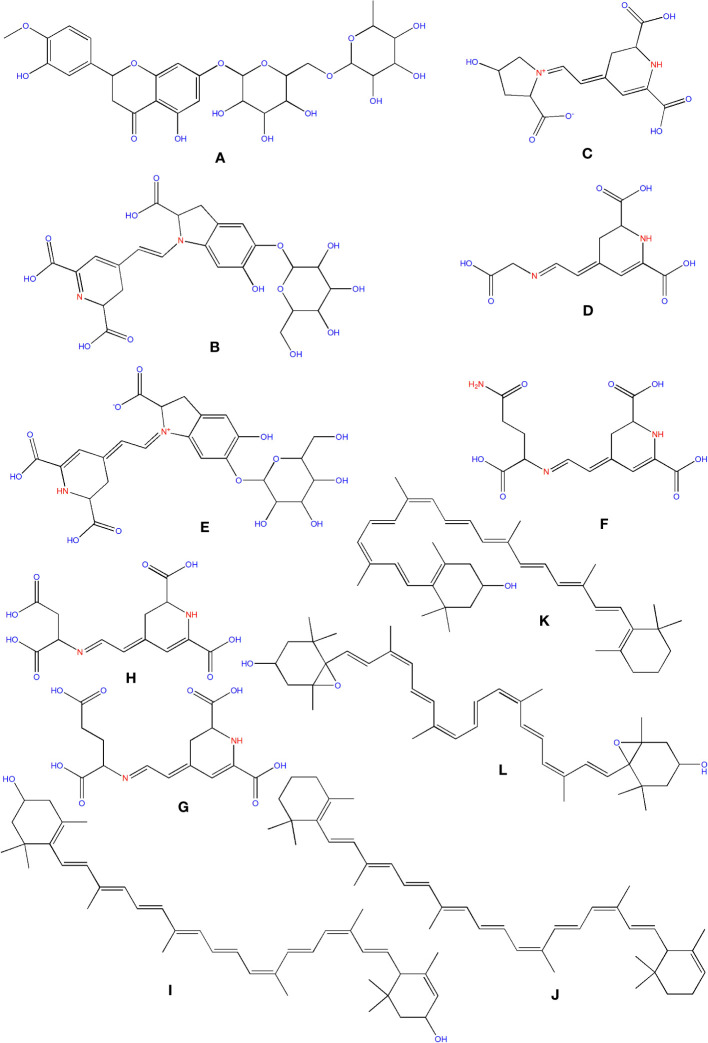
Structure of some of the phytochemicals isolated from *O. ficus-indica*. **(A)** Hesperidin; **(B)** Betanin; **(C)** Portulacaxanthin I; **(D)** Portulacaxanthin III; **(E)** Gomphrenin-I; **(F)** Vulgaxanthin I; **(G)** Vulgaxanthin-II; **(H)** Miraxanthin-II; **(I)** Lutein; **(J)** Carotene; **(K)** Cryptoxanthin; **(L)** Violaxanthin.

Flavanones, commonly called dihydroflavones, are a kind of flavonoid discovered in secondary metabolites of plants. In *O. ficus-indica*, three types of flavanones were found: naringenin (**Figure 6P**), naringin (**Figure 6Q**), and hesperidin (**Figure 9A**) (Zeghad et al., 2019; Hernández García et al., 2020). Hesperidin (16.18–17.46 mg/100 g), which was found mainly in cladodes, had the highest content, followed by naringin, mainly in peels (0.17 mg/100 g) (Belhadj Slimen et al., 2021). In peels, residues of naringenin were detected, while traces of naringenin were identified in fruits. *O. ficus-indica* peels have been verified as a flavanones source, whereas cladodes contain the highest hesperidin amount (Renugadevi and Prabu, 2009). More research is needed to evaluate these compounds in flowers and seeds. The glycosylated derivative of naringenin is naringin (C_27_H_32_O_14_), a polymethoxylated flavonoid (C_15_H_12_O_15_). The antioxidants naringenin and naringin are both potent (Jung et al., 2003).

### 7.2 Betalains

Betalains are nitrogen-containing hydro-soluble pigments. They are established in the sap of vacuoles and are mixed as bis-anions there (Brockington et al., 2015). Betalains were discovered in large quantities in the pulp and peel of *O. ficus-indica*. Peels have a higher concentration of betacyanins (19.43 compared to 6.76 mg/kg); however, fruits have a higher concentration of betaxanthins (53.27 compared to 40.72 mg/kg). Peels have more total betalains than fruits. Consequently, the peel’s betanin (**Figure 9B**) concentration is more significant than that of fruits (2473 mg/kg *vs*. 1616 mg/kg). The quantities of betacyanins and betaxanthins were closely related to the fruit coloring (Belhadj Slimen et al., 2021). De Wit et al. had compared seven cultivars of *O. ficus-indica* and one from *O. robusta*. Out of 7 cultivars, seeds of Nepgen (green) contains highest quantities of betacyanins (69.89 mg/Kg) and betaxanthins (48.92 mg/Kg), followed by seeds of Sicilian Indian Fig (pink) with betacyanins (56.50 mg/Kg) and betaxanthins (39.55 mg/Kg). However, seeds of *O. robusta* were having highest betacyanins and betaxanthins (de Wit et al., 2019). Further, in another study, De Wit et al. compared 4 cultivars of *O. ficus-indica* and one cultivar from *O. robusta*. Among various cultivars of *O. ficus-indica*, fresh fruit from Meyers cultivar possesses highest quantity of betacyanins (6.87 mg/kg) and betaxanthins (4.81 mg/Kg). However, again the fresh fruits from *O. robusta* contains betacyanins and betaxanthins, multifolds higher than that of *O. ficus-indica*. Their study also signifies that fresh and dried fruits are containing maximum betacyanins and betaxanthins, and it was significantly reduced in the form of chutney, juice, and preserves (De Wit et al., 2020). The peel also consists of muscaaurin, portulacaxanthin I (**Figure 9C**), portulacaxanthin III (**Figure 9D**), gomphrenin I (**Figure 9E**), (S)-valine-betaxanthin, (S)-isoleucine-betaxanthin, (S)-serine-betaxanthin, (S)-phenylalanine-betaxanthin, Vulgaxanthin I (**Figure 9F**), Vulgaxanthin II (**Figure 9G**), Vulgaxanthin IV, and miraxanthin II (**Figure 9H**) (Stintzing et al., 2005; Yeddes et al., 2013). Several investigations have identified betalains as an antioxidant dietary cationized type with a strong radical scavenging capacity (Kanner et al., 2001; de Wit et al., 2019; De Wit et al., 2020; Sigwela et al., 2021).

*In vitro* research has indicated that betalains elicit the phase II detoxifying enzyme quinone reductase in murine hepatoma cells (Madadi et al., 2020). Betalains actively scavenge free radicals, which may help to prevent cancer and cardiovascular disease. Betalains have been discovered to activate a critical factor of transcription, which causes to activate the endogenous defense of antioxidant systems in cells (Esatbeyoglu et al., 2014). In cancer cells, betalains from *Opuntia ficus-indica* fruits were discovered to have the effect of antiproliferative activity. In nude mice, betalains containing prickly pear fruit extracts inhibited the development of human ovarian cancer cells compared to a synthetic chemopreventive drug, Fenretinide (4-HPR). In addition, these extracts suppressed 40 to 60 percent of immortal cervical epithelial cells and cervical cancer cells (Zou et al., 2005). Betanin (betalain) was discovered to cause time and dose-dependent apoptosis in the leukemia cell line (K562) of human chronic myeloid, as well as a decrease in the potential of mitochondrial membrane and the presence of cyt C in the cytosolic portions of cells (Sreekanth et al., 2007). Subsequently, treatment with betalains and combining medication, which stops the process at several cell cycle points (G1 and/or S phases), can be a potential scheme for preventing cancerous tumor survival. Although the method by which betalains may prevent cancer is unknown, studies have indicated that they change gene expression and modulates cell development and apoptosis (Zou et al., 2005). Cancer cell lines like K562 and MCF7 enables researchers to investigate possible anticancer chemicals like betalains in a simple, controlled, and repeatable setting. However, because cell cultures cannot mimic tumor behavior and its interaction with the host (HogenEsch and Nikitin, 2012), the efficiency of betalains as an anticancer drug in human clinical trials may differ. Future research, combining cancer cell lines of humans within a model that profoundly resembles the targeted cancer of humans, will have a better chance of determining betalains’ anticancer potential (Gengatharan et al., 2015).

### 7.3 Carotenoids

**Figure 10:**
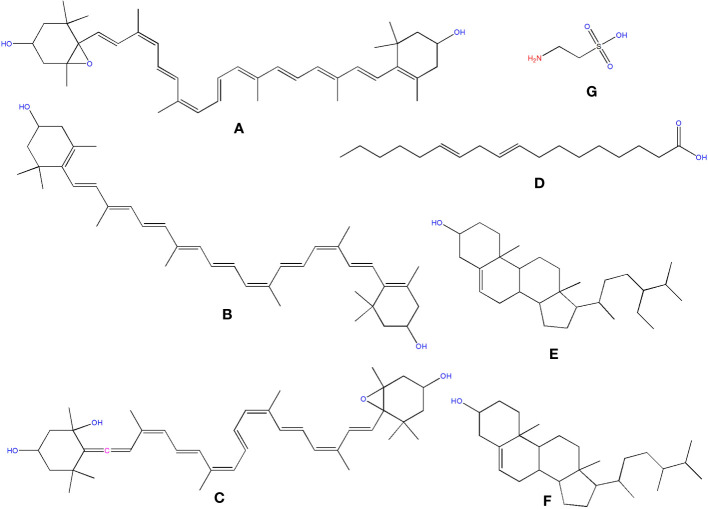
Structure of some of the phytochemicals isolated from *O. ficus-indica*. **(A)** Antheraxanthin; **(B)** Zeaxanthin; **(C)** Neoxanthin; **(D)** Linoleic Acid; **(E)** Sitosterol; **(F)** Campesterol; **(G)** Taurine.

Carotenoids are abundant in *O. ficus-indica*, and their concentration in the peel is substantially higher than in the pulp. Their maturation stage heavily influences the carotenoid composition of cladodes; the highly rich carotenoids in young cladodes are lutein (**Figure 9I**), carotene (**Figure 9J**), and β-cryptoxanthin (**Figure 9K**). Despite cultivar differences, cladodes carry more carotene than fruit (de Wit et al., 2019). Four carotenes and nine xanthophylls (84–86% of all carotenoids) make up the carotenoid profile of fruits. Lutein and violaxanthin (**Figure 9L**) were the most abundant xanthophyll compounds (representing 69–72 percent and 5 percent of all amount of carotenoid content in the whole fruit, respectively), whereas carotene was the most abundant pigment of carotene (representing almost 12–14 percent of the total carotenoid content). Lower concentrations of antheraxanthin (**Figure 10A**), zeaxanthin (**Figure 10B**), and neoxanthin (**Figure 10C**) have been found. The highest values were in orange-colored cultivars plants (Cano et al., 2017).

In 2014, Niranjana et al. examined carotenoids’ anticancer effectiveness through cell cycle arrest, apoptosis induction, anti-metastasis, and anti-angiogenic activities. One of the main characteristics of cancer cells is that they lose their ability to regulate the cell cycle and limit the proliferation rate. Carotenoids have been demonstrated to limit tumor cell proliferation by interfering with various cell cycle stages. Carotenoids have been shown to have chemopreventive properties in humans, lowering cancer incidence through apoptosis (Niranjana et al., 2015). Creating innovative anti-metastatic medications with minimal toxicity and excellent efficacy is one of the most active fields in contemporary anti-cancer research. Carotenoids have been shown in observational studies to have anti-metastasis properties (Kozuki et al., 2000). Various chemicals from natural sources have been proven to suppress angiogenesis, with carotenoids being particularly effective (Slimen et al., 2016).

Bensadón et al. determined the antioxidant and scavenging properties of polyphenols and carotenoids and other compounds by FRAP and ABTS methods in two varieties of *O. ficus-indica*, the Atlixco (52.22 ± 1.07 and 52.37 ± 2.00, respectively) and Milpa Alta (65.33 ± 2.23 and 57.55 ± 1.83, respectively) (Bensadón et al., 2010).

β-cryptoxanthin has shown its potential in various cancers, including stomach cancer (Sengngam et al., 2022), hepatocellular carcinoma (Lim et al., 2020), lung cancer (Iskandar et al., 2016), and colon cancer (San Millan et al., 2015). Lutein was reportedly having strong anticancer potential against breast cancer (Bulatao et al., 2023), non-small cell lung cancer (Zhang et al., 2023), pancreatic cancer (Han and Song, 2022), and gastric cancer (Eom et al., 2023). Zeaxanthin has anticancer potential against melanoma (Cenariu et al., 2021), colorectal cancer (Kim et al., 2019), dermal fibroblasts (Wu et al., 2010), and gastric cancer (Abnet et al., 2003).

### 7.4 Fatty acids

Chromatographic techniques were used to determine total lipids in various vegetative sections of *O. ficus-indica*. Linoleic acid (**Figure 10D**) has been identified as the most abundant cladodes polyunsaturated fatty acid, accounting for about 32.83 percent of the total fatty acid content. Palmitic acid, linolenic acid, oleic acid, and linoleic acid make up about 90% of cladode’s total fatty acids (Kolniak-Ostek et al., 2020). The polyunsaturated and mono-unsaturated fatty acid content in fruits ranged from 35.2% to 53.9% and 16.9% to 40.2%, independently, of total fatty acid content. Peels have lower concentrations of mono-unsaturated fatty acids than the concentration of polyunsaturated fatty acids.

Similarly, seed oil has a high proportion of polyunsaturated fatty acids but a low proportion of mono-unsaturated fatty acids. Linoleic acid can be considered the most common fatty acid in seed oil, fruits, and peels, which is interesting. Similarly, the content in peels is higher than that found in fruits (De Wit et al., 2017). Mono-unsaturated and polyunsaturated fatty acid consumption has been suggested for its health-improving qualities. They also help improve various health issues associated with cancer (Simopoulos, 2002).

### 7.5 Phytosterols

Sitosterol (**Figure 10E**) was found to be the most abundant sterol in oil fruit, seeds, peel, and pulp of *O. ficus-indica* (Kozuki et al., 2000), with amounts ranging from 6.75 to 21.1 g/kg. Campesterol (**Figure 10F**) was found in seeds, peels, and fruits in concentrations ranging from 1.66 to 8.76 g/kg. Other phytosterols discovered in trace amounts included stigmasterol, lanosterol, ergosterol, 5-avenasterol, and 7-avenasterol. Ergosterol (ergosta-5,7,22-trien-3-ol) is a sterol present in the cell membranes of fungi and protozoa. In animal cells, it performs the same functions as cholesterol. In human nutrition, ergosterol is a vitamin D_2_ provitamin. Remarkably, ergosterol was observed in Opuntia peels in small amounts. Phytosterols have been attributed to a reduced risk of breast, colon, and prostate cancers. Phytosterols, for example, have been found to enhance cancer immune recognition, impact hormonally dependent endocrine cancer growth, cycle arrest, and impede tumor growth and spread (Slimen et al., 2016).

### 7.6 Taurine

Taurine (C_2_H_7_NO_3_S) (**Figure 10G**) is an organic osmolyte not integrated into proteins. Taurine is an amino acid found in the brain, retina, muscles, and organs all over the body. Taurine is engaged in various processes, from development to cytoprotection, and is regarded as a cell-protective amino acid with antioxidative properties (Devamanoharan et al., 1998). The taurine content in cladodes, peels, and seeds should be examined. Taurine, the most abundant free amino acid, is involved in several biological processes in humans and has been found to have an antitumor effect (He et al., 2016). Kim and Kim examined the protective effects of taurine against anticancer medicines on normal cells, which were recently revealed. However, the anticancer effects of taurine on cancer cells are still unknown. As a result, we looked at the anti-cancer effects of taurine alone as well as taurine combined with cisplatin in human cervical cancer cells. In a time and dosage-dependent way, a single dose of taurine reduced cell growth. Cell proliferation was reduced more when cisplatin was combined with taurine than when cisplatin was used alone. Apoptosis induction resulted in decreased cell growth. Apoptotic cells were studied when cisplatin was treated with taurine. Taurine or cisplatin, in combination, promoted apoptotic cells more than taurine or cisplatin alone. The activation of caspase-3, caspase-6, caspase-7, and caspase-9 was attributed to the induction of apoptosis (Kim and Kim, 2013).

### 7.7 Saponins

Saponins are glycosides with triterpenoid or spirostane aglycones that have anti-mammalian disease pharmacological properties (Xu et al., 2016). More research on saponins in *O. ficus-indica* is needed. The presence of saponins in the aqueous extract of *O. ficus-indica* cladodes was discovered by Halmi and his associates (Halmi et al., 2018). Figueroa-Pérez and his colleagues found larger quantities in cladodes, averaging 28.13 g equivalents/kg (Figueroa-Pérez et al., 2018). Saponins show strong anti-tumorigenic effects through various anticancer mechanisms due to the considerable heterogeneity of their structures. Unique saponins with potent anticancer properties have also been developed. Ginsenosides, which belong to the dammaranes family, have been proven effective at inhibiting tumor angiogenesis by suppressing its inducer in blood vessel endothelial cells and preventing tumor cell adhesion, invasion, and metastasis. Dioscin, a steroidal saponin, and its aglycone diosgenin have indeed been researched extensively for their anticancer effects *via* cell cycle arrest and apoptosis (Man et al., 2010).

## 8 Future perspectives

**Figure 11:**
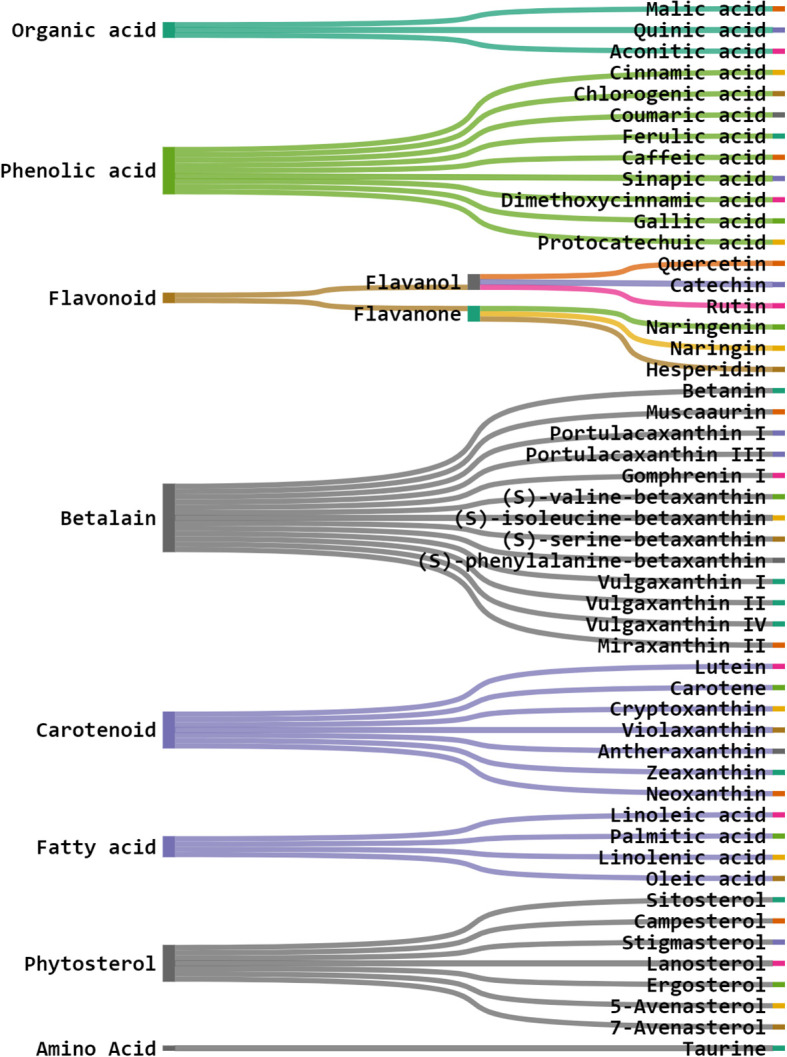
Phytochemicals present in *O. ficus-indica* and their phytochemical class.

*O. ficus-indica* contains various phytochemicals ranging from phenolic acids to phytosterols (**Figure 11**). Many phytochemicals like quercetin, hesperidin, and linolenic acid have already been evaluated and proven to have multifarious therapeutic potentials. However, it seems like more in-depth studies are required to validate the translational potential of *O. ficus-indica* to reach bedside.

**Figure 12:**
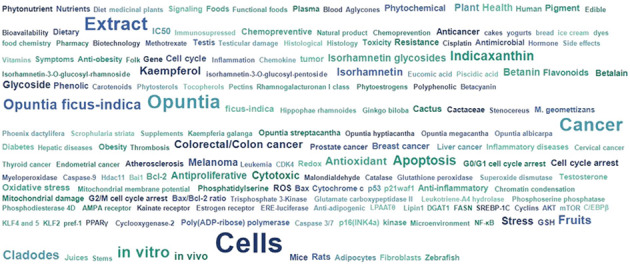
Quantitative data analysis-based study to understand the overall prevalence and dominating key terms in papers published with “*Opuntia ficus-indica*” and “Cancer”.

**Figure 13:**
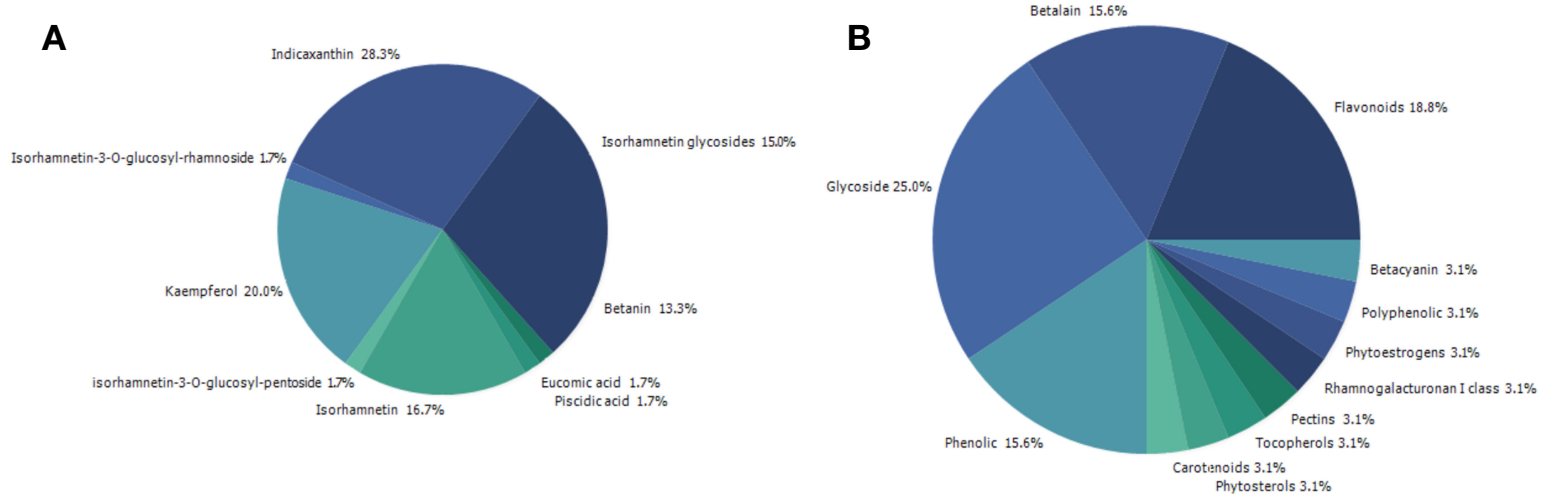
Quantitative data analysis-based study to understand the prevalence and dominating key terms in papers published with “*Opuntia ficus-indica*” and “Cancer.” **(A)** Prevalence of the studied phytochemicals; **(B)** Prevalence of the study’s phytochemical classes.

**Figure 14:**
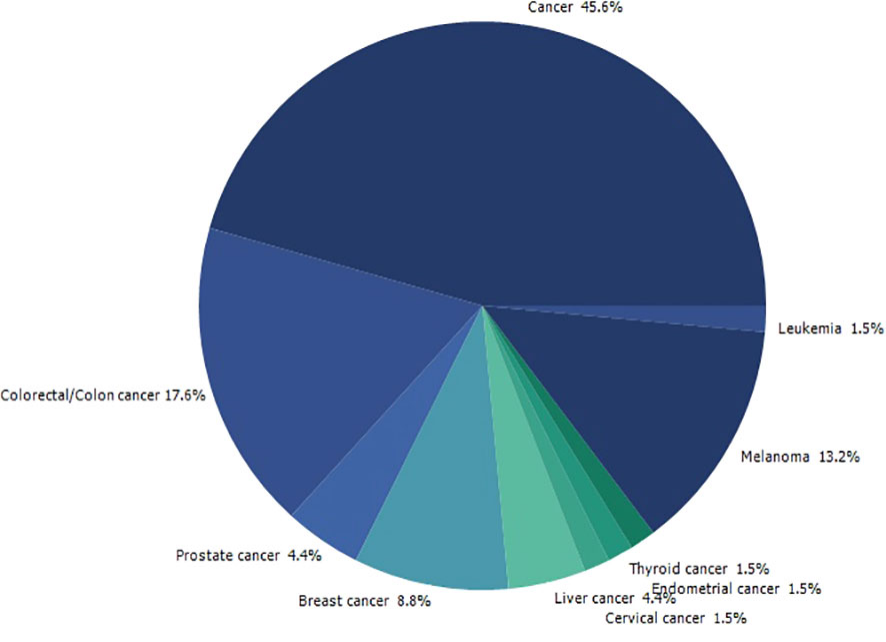
Quantitative data analysis-based study to understand the prevalence of the type of cancer being studied in papers published with “*Opuntia ficus-indica*” and “Cancer”.

**Figure 15:**
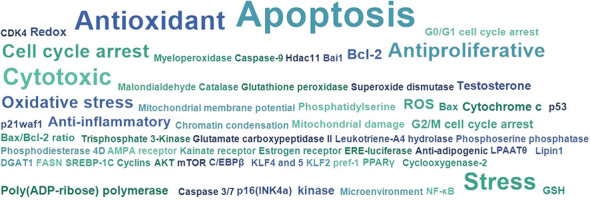
Quantitative data analysis-based study to understand the most studied and covered anticancer and related mechanisms in papers published with “*Opuntia ficus-indica*” and “Cancer”.

Searching on PubMed for *O. ficus-indica* and cancer-related papers yielded 18 results on 02.06.2023 while searching for *O. ficus-indica* and apoptosis-related papers resulted in 9 papers as of 03.06.2023. These studies indicate the increasing interest of researchers to examining anticancer potential of this plant. However, it is still quite low quantitatively and more in-depth research is utmost needed in this direction. Upon removal of duplicates, 21 articles were processed for quantitative data analysis (QDA). Some of the most prominent terminologies found to be “*Opuntia ficus-indica*,” “extract,” “indicaxanthin,” “kaempferol,” “glycoside,” “Opuntia,” “cancer,” “colorectal/colon cancer,” and “apoptosis.” (**Figure 12**) Indicaxanthin, kaempferol, and isorhamnetin glycosides were most studied as anticancer agents in those studies (**Figure 13A**). When we looked for the prevalence of the phytochemical class mentioned repeatedly in those 21 articles, we had observed that glycosides dominate, followed by flavonoids and betalains (**Figure 13B**). Most anticancer studies on *O. ficus-indica* and its purified metabolites are related to colorectal/colon cancer, followed by melanoma and breast cancer (**Figure 14**). Very little attention has been paid to leukemia, thyroid, endometrial, liver, and prostate cancer, and it should be considered an opportunity for the researchers to explore *O. ficus-indica* and its metabolites for these heterogenous cancers. The most notable mechanisms expressed and validated in those studies were apoptosis, cell cycle arrest (G0/G1 and G2/M), Bcl-2 modulation, antiproliferative, oxidative stress-mediated mechanisms, and cytochrome c (**Figure 15**). We have also observed that cladodes and fruits of *O. ficus-indica* have been more studied than other plant parts, which again opens opportunities for researchers to explore. Further, cell line-based studies dominated, and there were very few studies related to animal-based experiments. The Zebrafish model has also been utilized by one group, and it could open a new platform for others to explore.

## 9 Conclusion

In this article, we discussed the effectiveness of *O. ficus-indica* against most death-causing diseases, including cancer. Traditional chemo- and radiotherapies can weaken the patient due to their toxic and destructive effects on normal cells. The *O. ficus-indica* plant is a natural means that can be used for the prevention and cure of cancer without any serious side-effects. Various phytochemicals are present in *O. ficus-indica*, which includes flavonoids, polyphenols, betalains, and tannins, which possess various biological activities such as antimicrobial, anti-inflammatory, antioxidant, and most essential, anticancer activity. Based on the available literature we provided in this study, investigations using diverse cancer cell lines have shown compelling evidence of anticancer activity. Only a few investigations employing *in vivo* tumor models have produced comparable results. Currently, very little information is available on whether concentrations demonstrating activity *in vitro* are attainable in the blood or serum of laboratory animals. While this is a restriction in our current knowledge of the anticancer potential of plants in the *Cactaceae* family, future studies are likely to examine this to next level. We believe that the significance of our review lies in drawing the scientific community’s attention to this plant family, which is, on the one hand, auspicious in terms of cancer treatment and prevention, but on the other hand, remains mostly unexplored.

## Author contributions

JW, NR, SJ, RR, SK, SaK, SvK, BD, JS-G, BS, and RKS wrote different manuscript sections. NR, RKS, and BS laid down the idea and manuscript outline and compiled and finalized the manuscript. BS arranged the funding source. All the authors have reviewed the final version of the manuscript.
